# Primates in Alcohol Research

**Published:** 1995

**Authors:** J. Dee Higley

**Affiliations:** J. Dee Higley, Ph.D., is a research psychologist at the National Institute on Alcohol Abuse and Alcoholism, Laboratory of Clinical Studies, Primate Unit, Poolesville, Maryland

**Keywords:** animal model, AOD dependence, research, hereditary factors, environmental factors, Cloninger’s typology

## Abstract

The genetic similarity to humans of nonhuman primates makes them well suited to serve as models of complex human disorders such as alcoholism. Like humans, nonhuman primates vary with respect to their alcohol consumption, even within the same species. Studies of the origins of high consumption among nonhuman primates have suggested that both genetic and environmental factors play a role in their drinking. In fact, researchers have found some support for multiple subtypes of alcoholism among nonhuman primates.

Because of their close genetic similarity to humans and their complex social behaviors, nonhuman primates have been widely used to study a number of psychiatric syndromes (for a review, see McKinney 1988). Although they have been used less often in alcohol research, nonhuman primates are ideally suited as subjects for psychobiological studies of excessive alcohol consumption, because the similarities in human and other primate DNA result in their sharing many physiological and behavioral processes. In addition, nonhuman primates have genetic and rearing backgrounds that can be carefully planned so as to allow for observation in closely controlled settings. These two attributes allow researchers to extrapolate results from studies of nonhuman primates to humans more readily than the results from less closely related animals.

This article reviews examples of ongoing research that uses non-human primates to investigate how genetic predispositions influence alcohol consumption. It also examines how a genetic predisposition may interact with other factors, such as environment and exposure to stress, to produce greater voluntary alcohol consumption.

## Primate Drinking

One reason nonhuman primates are not used frequently in alcohol research is that they rarely consume alcohol in the amounts necessary to create models of human alcoholism. Some researchers have suggested that this is because the animals find the initial taste of alcohol aversive (Higley et al. in press *b*). Most humans also initially find the taste of alcohol unpleasant, particularly when the alcohol concentration in a solution is high. Consequently, alcohol is rarely consumed in its pure state. Instead, it is consumed in solutions with low concentrations; the taste often is disguised using colas, fruit juices, and other flavorings. Similarly, in at least some species of nonhuman primates, when the solution is palatable and the concentration of alcohol is under 15 to 20 percent, most animals will consume alcohol at rates producing pharmacological effects. Only about 10 to 20 percent of subjects who have not been subjected to stressful environmental conditions that induce drinking, however, will freely consume palatable alcohol solutions at rates that consistently produce blood alcohol levels greater than the “legal” level of intoxication for most States (Ervin et al. 1990; Higley et al. in press *b*; Kraemer and McKinney 1985).

Because few animals consume alcohol at rates higher than the legal intoxication levels, when using nonhuman primates, such as monkeys, researchers must have access to a sizable population of subjects. The cost of maintaining nonhuman primates prevents most research facilities from supporting populations large enough to study excessive alcohol consumption.

Most nonhuman primates that have been tested for alcohol preference are Old World species (e.g., baboons, vervet monkeys, and many macaques), although at least one study investigated squirrel monkeys, a New World species that frequently is used in the laboratory (Kaplan et al. 1981). Although consumption rates vary somewhat between species, when an alcohol solution is palatable and freely available, most Old World species consume alcohol in amounts that produce pharmacological effects. Nevertheless, wide differences exist between individuals within a species. However, as discussed below, other factors can increase the amount of alcohol that individual subjects consume.

## Studies of Individual Differences

Researchers studying alcohol consumption in nonhuman primates have made significant progress in developing an animal model of alcohol abuse^1^ by focusing research on subjects that show high rates of voluntary alcohol consumption. Studies show that once primates’ rates of consumption stabilize, average differences in consumption between subjects are markedly consistent over time (Higley et al. 1991; Kraemer and McKinney 1985). A similar phenomenon has been observed in humans. This traitlike alcohol-consumption pattern indicates the potential for nonhuman primates to model the etiological mechanisms of high alcohol preferences.

### Applying Subtypes to Nonhuman Primates

Researchers investigating alcohol problems now widely recognize that alcohol abuse and alcoholism have multiple etiologies (Cloninger 1987; Gilligan et al. 1987). Thus, categorizing alcoholism into subtypes has developed as a method for recognizing patterns in the symptoms and causes of the disorder. Cloninger delineated two of the best known subtypes (Cloninger 1987). The first subtype, anxiety-related alcoholism, Cloninger labeled as type I. This subtype typically is less severe than the second subtype, develops during adulthood, and has been associated with adverse experiences in childhood. The second subtype, which Cloninger labeled type II alcoholism, is characterized by aggression, antisocial behavior, and the onset of alcohol problems early in life. Both subtypes appear to be influenced by inherited, or genetic, factors. Nonhuman primates have only recently begun to be used to investigate the underlying mechanisms that produce type II alcoholism. They have been employed with increasing frequency, however, in studies of the type I, or anxiety-related, disorder.

#### Research Methods

Studies of the factors that affect drinking rates in nonhuman primates often involve comparing subjects exposed to stressful environments during infancy with those raised under normal laboratory conditions. For example, rhesus macaques that spend the first 6 months of their lives only in the constant company of their age peers (i.e., peer reared) and are chronically absent from their mothers behave differently, displaying less curiosity and more fear, than those raised by their mothers (i.e., mother reared). In adolescence and in adulthood, drinking rates in the two groups can be compared during additional stress-provoking challenges, such as separating subjects from other monkeys for certain periods of time (i.e., social separation) (Higley et al. 1991). The macaques’ responses to the challenges differ, depending at least in part on their rearing experiences (discussed below).

##### Type I

**Figure 1:**
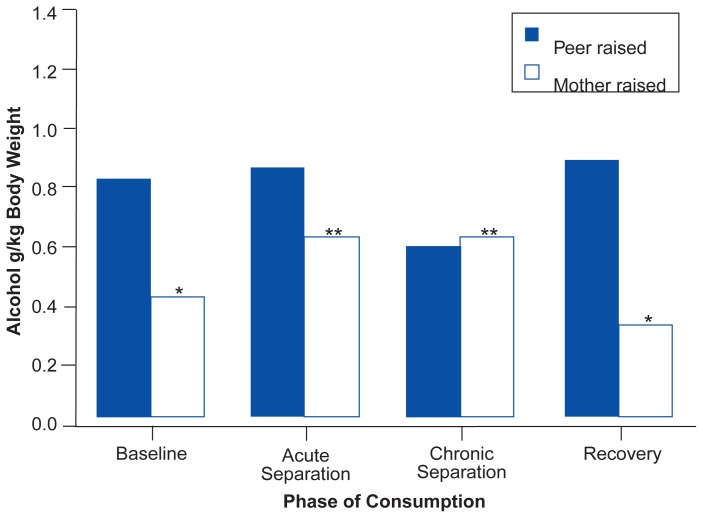
Effects of early rearing experiences and social separation on alcohol consumption in adolescent rhesus macaques (*N* = 22). The baseline bar shows the average consumption over 10 days before separation from other group members. The other bars represent the average consumption for each group during and after periods of separation. The average consumption during separation is divided into an acute phase (mean consumption on the first day of each separation) and a chronic phase (mean consumption on the remaining 3 days of each separation). The recovery bar shows the average of 10 days of alcohol consumption postseparation. * = significant difference between the peer-reared subjects and the mother-reared subjects within the same period. ** = significant increase in alcohol consumption among the mother-reared subjects during social separation compared with consumption before separation. The apparent reduction in alcohol consumption by the peer-reared monkeys during the chronic phase of the social separation is not statistically significant. SOURCE: Adapted from Higley et al. 1991.

Studies using adolescent rhesus macaques have shown that during nonstressful periods, such as routine life in subjects’ home environments, individual rates of alcohol consumption are positively correlated with characteristics of anxiety and fearfulness among animals (Higley et al. 1991). Such characteristics may be either learned or inherited. For example, these studies show that early rearing experiences placing subjects at risk for high levels of anxiety, such as depriving an infant of its parents, may increase alcohol consumption. In fact, the majority of rhesus macaque subjects that are reared in peer-only groups consume alcohol during nonstressful periods at rates producing average blood alcohol concentrations that exceed most legal intoxication limits (Higley et al. 1991). High rates of alcohol consumption also can be induced in many mother-reared subjects by increasing the amount of stress to which they are subjected. For example, in response to a social-separation stressor, mother-reared adolescent subjects increase their rates of alcohol consumption to match the rates of peer-reared subjects (figure 1) (Higley et al. 1991; Kraemer and McKinney 1985).

Nevertheless, not all mother-reared macaques increase their alcohol consumption rates in response to such stressors, and some peer-reared subjects do not develop high consumption rates following their early rearing experiences. These animals also display lower levels of anxiety, and their moderate responses may result from genetic predispositions. Within both the peer- and the mother-reared groups, individual differences in alcohol consumption are directly related to behavioral measures of anxiety, such as clasping themselves or behaviorally withdrawing, and biological measures, such as levels of plasma corticotropin, a hormone secreted by the pituitary gland that increases during periods of most types of stress. These findings suggest that increased levels of anxiety, whether resulting from prior early rearing experiences or from temporary challenges such as social separation, can increase alcohol consumption. These results are similar to those observed in Cloninger’s type I alcoholism (Cloninger 1987).

##### Type II

Higley and colleagues recently have begun a series of studies designed to investigate parallels between Cloninger’s type II alcoholism and correlates of high alcohol consumption in adolescent and young adult rhesus macaques living in groups. These studies have been reported in preliminary form (Higley et al. 1994; King et al. 1993).

The findings show that high rates of alcohol consumption are observed in subjects with infrequent social interactions, less competent social behaviors, and high rates of aggression. These subjects are violent, impulsive, and eventually ostracized by their peers (Higley et al. 1993; Mehlman et al. 1994). Their antisocial-like characteristics appear to be related in part to early rearing experiences. Subjects that are peer reared show impaired functioning of the brain chemical serotonin (as measured by cerebrospinal fluid [CSF] concentrations of 5-hydroxyindoleacetic acid [5–HIAA], a product of serotonin metabolism) beginning early in infancy and continuing into adulthood, suggesting that 5–HIAA levels are influenced by rearing experiences. Serotonin is believed to help control impulses and mood. Adult peer-reared subjects have low CSF 5–HIAA concentrations and exhibit inept social behaviors. Frequently, they must be removed from their social groups for excessive aggression and deviant behavior.

Impaired serotonin function also results from genetic background (Higley et al. 1993), and some subjects show impaired behavior regardless of rearing experience. Within both mother-and peer-reared groups, subjects with low CSF 5–HIAA concentrations exhibit reduced rates of social interaction, low social dominance rankings, and high rates of violence (Higley et al. in press *a*) and alcohol consumption (King et al. 1993). The increased alcohol consumption, inappropriate aggression, and incompetent social behaviors seen in adolescent and adult nonhuman primate subjects with low levels of CSF 5–HIAA parallel findings in human alcoholics with low serotonin functioning (Virkkunen et al. 1994*a*,*b*).

With some exceptions, these findings generally are consistent with predictions from Cloninger’s type II model of excessive alcohol consumption in men, among whom Cloninger has found low CSF 5–HIAA levels (Cloninger 1987; Virkkunen et al. 1994*a*,*b*). Because the nonhuman primates that exhibit type II features of alcoholism also have high levels of anxiety, these findings also suggest a partial overlap between type I and type II alcoholism, an intimation that may merit further study in humans.

## Genetic Influences

**Figure 2:**
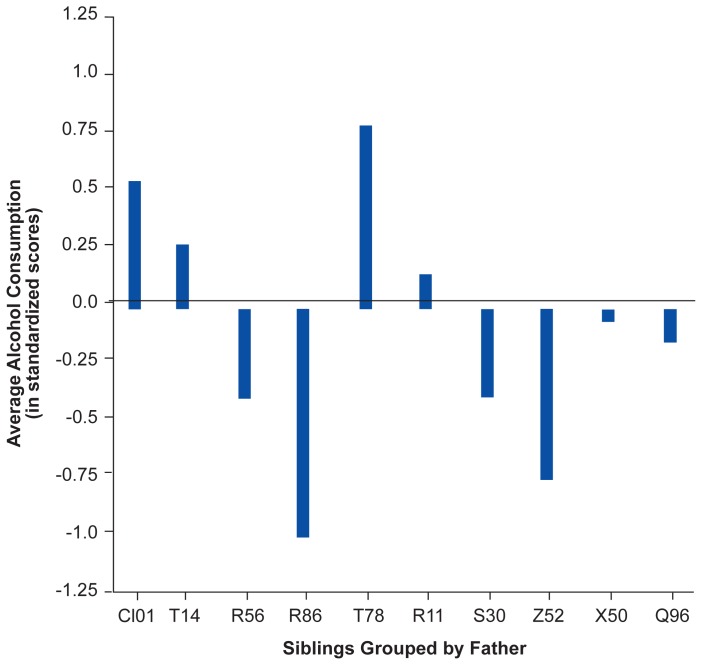
Alcohol consumption for 10 groups of rhesus macaques (26 males and 29 females) reared apart from their fathers. Each group (represented by a bar) includes at least three members, all of whom have the same father. For comparison, the “0.0” line represents the average consumption for a typical group of rhesus macaques. Thus, the chart shows that offspring of father T78 consistently consume more alcohol than the average, whereas offspring of father R86 consume less. The figure demonstrates that alcohol consumption may indeed be a product of paternal genetic influences.

Results of the type I and type II studies using nonhuman primates have hinted that genetic influences play a role in rates of alcohol consumption. Emerging evidence from nonhuman primate sibling studies also suggests a genetic influence on alcohol consumption in rhesus macaques. Rates of alcohol consumption were studied in 55 paternal half-sibling adolescent and young adult subjects that were reared apart from their fathers. Although the results are preliminary because of the small sample size, more than 50 percent of the variation between subjects in rates of alcohol consumption appeared to result from paternal genetic contributions, suggesting that genetic factors greatly influence alcohol intake rates. Figure 2 shows the average rates of consumption grouped statistically by father. Higley and colleagues currently are investigating genetic-environmental interactions and maternal genetic contributions to alcohol intake in the rhesus macaques. By selectively breeding subjects for similar neurogenetic traits, such as low CSF 5–HIAA, and rearing them in different highly controlled environments, Higley and colleagues plan to investigate the observed behaviors (i.e., phenotypes^2^) and underlying genetic traits that lead to excessive alcohol consumption.

## Efficacy as Models

Although nonhuman primates with high rates of alcohol consumption have been shown to exhibit aspects of type I and type II alcoholism, a number of features of alcoholism are yet to be demonstrated adequately in these animals to produce a model for all aspects of alcoholism. For example, in contrast to human data (Cloninger 1987), Higley and colleagues (in press *b*) found no gender differences in rates of alcohol consumption among rhesus macaques. In addition, long-term voluntary consumption at rates that produce physiological withdrawal and prolonged social deficits—such as failure to parent adequately or to maintain social bonds—has not been demonstrated routinely in nonhuman primates.

## Conclusions

A phenotype for high rates of alcohol consumption is present in at least some species of nonhuman primates. Subjects that exhibit this behavior will consume alcohol at rates producing intoxication. Adverse experiences early in life appear to exaggerate genetic predispositions for alcohol consumption. Underlying etiological mechanisms and biobehavioral correlates of the high-consumption phenotype—such as stress, anxiety, antisocial-like behaviors, and impaired serotonin functioning—parallel many of the predictions of Cloninger’s neurogenetic model of alcoholism.

These examples from nonhuman primate research demonstrate its value for studying aspects of human alcoholism. Thus, it is surprising that nonhuman primates have been used so infrequently in this field. Their use in alcohol consumption research is a relatively recent development. Fewer than 100 research papers on this topic have been published during the past 25 years, compared with the more than 380 articles published over the past 3 years alone on alcohol consumption in mice and rats. Because humans and other primates share a large percentage of their genetic material (Stone et al. 1987), studies of nonhuman primates will help clarify the genetic and environmental interactions that contribute to the development of alcoholism in humans.

## Footnotes

1 In this article, the term “alcohol abuse” refers to patterns of problem drinking that have resulted in adverse effects on health, social functioning, or both. The term “alcoholism” refers to abnormal alcohol-seeking behavior that leads to impaired control over drinking.

2 For a definition of this and other technical terms used in this article, see central glossary, pp. 182–183.
